# The Progression of *NUS1* ‐Associated Parkinson's Disease and the Diagnostic Potential of Plasma NgBR

**DOI:** 10.1111/cns.70549

**Published:** 2025-07-28

**Authors:** Lizhi Li, Juanjuan Huang, Yaqin Xiang, Xuxiang Zhang, Qian Xu, Qiying Sun, Zhenhua Liu, Xinxiang Yan, Jinchen Li, Beisha Tang, Jifeng Guo

**Affiliations:** ^1^ Department of Neurology, Xiangya Hospital Central South University Changsha Hunan China; ^2^ Department of Radiology Xiangya Hospital, Central South University Changsha Hunan China; ^3^ Department of Geriatrics, Xiangya Hospital Central South University Changsha Hunan China; ^4^ National Clinical Research Centre for Geriatric Disorders, Xiangya Hospital Central South University Changsha Hunan China; ^5^ Key Laboratory of Hunan Province in Neurodegenerative Disorders Central South University Changsha Hunan China; ^6^ Hunan Key Laboratory of Medical Genetics, School of Life Sciences, Centre for Medical Genetics Central South University Changsha China; ^7^ Hunan International Scientific and Technological Cooperation Base of Neurodegenerative and Neurogenetic Diseases Changsha China

**Keywords:** disease progression, Nogo‐B receptor, *NUS1* gene, Parkinson's disease

## Abstract

**Aims:**

The study aimed to investigate the role of *NUS1* variants in Parkinson's disease (PD) progression and evaluate plasma Nogo‐B receptor (NgBR) as a potential biomarker.

**Methods:**

We recruited 228 PD patients, including 38 with *NUS1* variants (*NUS1*‐PD) and 190 without (GU‐PD), and all underwent at least two follow‐up visits. Linear mixed‐effects models assessed motor and non‐motor symptom progression. Plasma NgBR levels were measured in PD, Multiple System Atrophy (MSA), Progressive Supranuclear Palsy (PSP), and healthy controls (HC), and receiver operating characteristic curve analysis evaluated its diagnostic efficacy.

**Results:**

*NUS1*‐PD demonstrated an earlier age at onset and more severe motor features than GU‐PD at baseline. Longitudinal analyses showed similar progression rates of UPDRS III and H&Y stage (off‐medication) in *NUS1*‐PD and GU‐PD, but a slower progression rate of urinary function in *NUS1*‐PD (*p* = 0.024). Plasma NgBR levels were higher in PD than in HC, MSA, and PSP, with AUC values of 0.6832, 0.6716, and 0.6628, respectively. Plasma NgBR was associated with UPDRS III (*p* = 0.006) and cognitive impairment (*p* = 0.010).

**Conclusion:**

*NUS1* variants show no impact on PD progression, while plasma NgBR may serve as a potential biomarker for PD diagnosis and clinical characteristics.

## Graphical abstract

The study aimed to investigate the role of *NUS1* variants in PD progression and evaluate plasma Nogo‐B receptor (NgBR) as a PD biomarker. We found that *NUS1* variants show no impact on PD progression, while plasma NgBR is a potential biomarker for PD diagnosis and clinical characteristics.

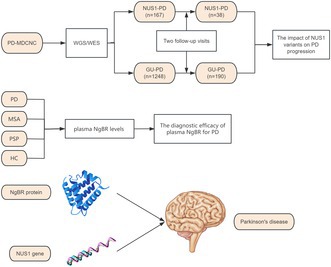

## 1 Introduction

Parkinson's disease (PD) is a progressive neurodegenerative disorder characterized by a spectrum of motor features encompassing static tremor, rigidity, bradykinesia, and postural instability. PD also manifests with a range of non‐motor symptoms, such as hyposmia, depression, cognitive dysfunction, and daytime sleepiness [1]. Neuropathological hallmarks of PD include the loss of dopaminergic neurons in the substantia nigra and the formation of Lewy bodies (LB), which are composed of alpha‐synuclein (a‐syn) aggregates. Given the obstacle in detecting the pathological characteristics of PD, the diagnosis of PD currently relies predominantly on clinical features [2]. Moreover, several studies have underscored the utility of various biomarkers, including biofluid‐based biomarkers, imaging biomarkers, and microRNAs, in improving diagnostic precision [3, 4].

The etiology of PD remains incompletely elucidated. It is reported that genetic and environmental factors are jointly involved in the onset of PD. Notably, *SNCA*, *LRRK2*, *GBA*, and other genes have been implicated in the pathogenesis of PD [5]. Furthermore, our previous study demonstrated for the first time that rare coding variants of *NUS1* were associated with PD [6]. Subsequent studies conducted by our team provided additional evidence supporting the association between both coding and non‐coding variants of *NUS1* and PD pathogenesis [7, 8]. However, the role of the *NUS1* gene in PD remains controversial, and some studies have not identified a significant contribution of *NUS1* variants to PD risk [9, 10, 11]. Besides, animal models with *NUS1* knockdown exhibit both pathological and clinical features of PD [6, 12].

*NUS1* gene encodes Nogo‐B receptor (NgBR), which is mainly located in plasma membranes and endoplasmic reticulum (ER). NgBR is involved in promoting vascular remodeling and formation, regulating cholesterol metabolism, protein N‐glycosylation, and the synthesis of Dolichol phosphate [13, 14, 15]. NgBR exerts physiological functions in the central nervous system (CNS) via the KAT7/RFX1/FGF1 axis [16]. Besides, NgBR deficiency in Drosophila and zebrafish models has been shown to cause cholesterol accumulation in the brain, leading to loss of dopaminergic neurons, increased a‐syn neurotoxicity, and movement abnormalities [12, 15].

According to previous research, PD associated with *LRRK2* and *GBA* has distinctive clinical phenotypes and varying disease progression rates [17, 18]. Similarly, it is reported that PD patients harboring *NUS1* variants have an earlier age at onset (AAO) and increased susceptibility to developing psychiatric symptoms [8]. However, no systematic studies have yet elucidated the clinical characteristics and disease progression rates of *NUS1*‐associated PD. In addition, PD patients carrying the *NUS1* c.691 + 3dupA variant show significantly decreased mRNA expression, but no studies have investigated the changes in NgBR levels in either plasma or cerebrospinal fluid (CSF) [6]. Given the clinical utility and accessibility of peripheral biomarkers in neurodegenerative diseases, the evaluation of plasma NgBR may offer valuable insights. Therefore, this study aims to investigate the clinical features of *NUS1*‐associated PD through longitudinal follow‐up studies and delineate the role of plasma NgBR in PD.

## 2 Methods

### 2.1 Study Design and Participants

All participants were recruited from Xiangya Hospital, Central South University, and other collaborative institutions of the Parkinson's Disease and Movement Disorders Multicenter Database and Collaborative Network in China (PD‐MDCNC, http://pd‐mdcnc.com/). Before enrollment, participants signed informed consent. The diagnosis of PD was based on the clinical diagnostic criteria of the United Kingdom Parkinson's Disease Society Brain Bank [19] or the International Parkinson and Movement Disorders Society (MDS) [20]. PD patients who received surgical treatment, including deep brain stimulation (DBS), were omitted from the study. Additionally, Progressive Supranuclear Palsy (PSP) diagnosis adhered to the 2017 MDS diagnostic criteria [21], and the diagnosis of Multiple System Atrophy (MSA) was established following the diagnostic consensus statement formulated by S. Gilman [22]. Two neurologists confirmed the diagnosis, and participants without neurological diseases were included as healthy controls (HC). Baseline information collection and scale assessment were conducted on all enrolled participants, and 10 mL of blood was collected using EDTA anticoagulation tubes.

### 2.2 Genetic Testing

The PD patients carrying *NUS1* nonsynonymous variants in the study were derived from three previous studies conducted by our team [6, 7, 8]. The cohort of PD patients with *NUS1* variants was named *NUS1*‐PD. The remaining participants (both patients and healthy controls) underwent Whole Exome Sequencing (WES) or Whole Genome Sequencing (WGS), and the specific steps were performed as described previously [23, 24]. Cohorts including PD patients without *NUS1* variants, MSA patients, PSP patients, and healthy controls were named GU‐PD, MSA, PSP, and HC, respectively. Notably, all participants in the GU‐PD, MSA, PSP, and HC did not carry known PD pathogenic genes or *NUS1* variants.

### 2.3 Clinical Assessment

All baseline and follow‐up data of enrolled participants are saved in PD‐MDCNC. At baseline, comprehensive information of all participants, including demographic information, medical history, lifestyle habits, and scale assessments, was collected. Subsequently, pertinent information regarding medication utilization, disease progression, and scale assessments was documented during follow‐up visits. The disease duration of levodopa therapy initiation was recorded, and the levodopa equivalent daily dose (LEDD) at each visit was calculated in accordance with formula [25]. All participants completed baseline data collection, but only PD patients were followed up and included in the longitudinal studies.

The evaluation scales of PD symptoms included the Unified Parkinson's Disease Rating Scale (UPDRS), Hoehn and Yahr (H&Y) stage, the Non‐Motor Symptoms Scale (NMSS), the Mini‐Mental State Examination (MMSE), Parkinson's Disease Sleep Scale (PDSS), Epworth Sleepiness Scale (ESS), RBD Questionnaire‐Hong Kong (RBDQ‐HK), Hyposmia Rating Scale (HRS), Parkinson Fatigue Scale (PFS), Scale for Outcomes in PD for Autonomic Symptoms (SCOPA‐AUT), Cambridge‐Hopkins questionnaire for restless legs syndrome (CH‐RLSq), Rome III: Diagnosis criteria for IBS, PD Questionnaire‐39 (PDQ‐39), and the 17‐item Hamilton Depression Rating Scale (HAMD). Besides, the data on levodopa‐induced dyskinesias (LID) and freezing of gait (FOG) were also documented. UPDRS is composed of four distinct sections to assess different symptoms of PD: non‐motor symptoms (UPDRS I), activities of daily living (UPDRS II), motor examination (UPDRS III), and therapy‐related complications (UPDRS IV). We further calculated the scores of motor symptoms, including tremor (UPDRS items 20 and 21), rigidity (item 22), bradykinesia (items 23–26 and 31), and axial symptoms (items 27–30) based on UPDRS. The ratio of tremor score and axial symptoms score was computed to classify PD patients as tremor‐dominant (TD), indeterminate, and postural instability and gait difficulty (PIGD) [26]. The evaluations of UPDRS III and H&Y stage were completed after withdrawing anti‐parkinsonian medications for at least 12 h (off‐medication). Additionally, cognitive impairment was defined according to the MMSE scoring criteria: a score below 17 for illiterate individuals, a score below 20 for those with ≤ 9 years of education, and a score below 24 for individuals with > 9 years of education.

### 2.4 Follow‐Up Plan

Specifically, recruited PD patients were interviewed through face‐to‐face visits every 2 years, and UPDRS III and H&Y stage were assessed in “off‐medication”. All PD patients with at least two follow‐up visits were included in the longitudinal study.

### 2.5 Measurement of Plasma NgBR

Plasma was obtained from blood by centrifugation at 3000 rpm for 10 min at 4°C and stored in −80°C refrigerators. Plasma NgBR levels were measured using enzyme‐linked immunosorbent assay (ELISA) according to the protocol. Each plasma sample underwent two rounds of measurement, and if the coefficient of variation exceeded 15%, a remeasurement was conducted. To evaluate the diagnostic efficacy of plasma NgBR levels in distinguishing PD, we incorporated *NUS1*‐PD, GU‐PD, HC, MSA, and PSP.

### 2.6 Statistical Analyses

The Kolmogorov–Smirnov test and the P–P plot were employed to assess the normality of the distribution. Between‐group comparisons were performed using Student's t‐test, one‐way ANOVA, and Mann–Whitney *U*‐test for continuous variables, while categorical variables were analyzed using the chi‐square test and Fisher's exact test. Multivariable linear regression was applied to adjust for confounding factors, including AAO, disease duration, disease duration at levodopa therapy initiation, LEDD, years of education, and gender when analyzing baseline data. Moreover, multivariable linear regression and logistic regression were performed to adjust for age, gender, and plasma storage time when investigating the association between plasma NgBR levels and PD. The diagnostic and differential diagnostic capabilities of plasma NgBR levels for PD were assessed by receiver operating characteristic (ROC) curves. Kaplan–Meier survival analysis and Cox proportional hazards models were used to ascertain the survival rates and hazard ratios (HR) of FOG over time, adjusting for variables including gender, AAO, and disease duration at levodopa therapy initiation at baseline. Additionally, linear mixed‐effects models were utilized to evaluate the progression rates of motor and non‐motor symptoms, with random intercept and random slope models tested. Linearity of models was assessed by plotting residuals against predicted values, and Q‐Q plots was used to evaluate the normality of residuals. The covariates of motor symptom analysis included age, gender, LEDD, and years of education at baseline. In addition to the above, UPDRS III (off‐medication) was incorporated into the analysis of non‐motor symptoms. *p* values were adjusted using the Bonferroni correction, and results with *p <* 0.05 but not passing the Bonferroni correction were considered suggestively significant. All statistical analyses were conducted by IBM SPSS Statistics (version 27).

## 3 Results

### 3.1 Baseline Demographic and Clinical Profiles

At baseline, the demographic and clinical characteristics of 768 GU‐PD and 112 *NUS1*‐PD are shown in Table 1. The results showed significant differences between *NUS1*‐PD and GU‐PD in AAO and disease duration of levodopa therapy initiation. Moreover, at baseline, UPDRS total score, UPDRS II, UPDRS III, and three motor symptom scores (tremor, rigidity, and PIGD) of *NUS1*‐PD were significantly higher than those of GU‐PD. Additionally, H&Y stage was notably higher in *NUS1*‐PD compared to GU‐PD. After adjusting for confounding factors, the differences in UPDRS and H&Y stage between *NUS1*‐PD and GU‐PD remained (Table S1). We also detected suggestive differences between the two groups in the incidence of FOG, D3 of NMSS (mood/apathy), and PDQ‐39, but none of these differences remained significant after the Bonferroni correction.

**TABLE 1:** Demographic and clinical characteristics of PD patients at baseline.

	GU‐PD (*n* = 768)	*NUS1*‐PD (*n* = 112)	*p*
Demographics			
Male (%)	52.08%	59.82%	0.125
Year of education, y	10 (6, 12)	9 (6, 12)	0.237
Age at onset, y	54 (47, 61)	48 (42, 57)	< 0.001*
Age at baseline, y	58 (51, 65)	54.5 (46.25, 63.5)	0.003
Disease duration, y	2 (1, 5)	3.5 (1, 7)	0.003
Disease duration at levodopa therapy initiation, y	1 (0, 2)	1 (0.25, 3)	< 0.001*
LEDD, mg/24 h	300 (150, 450)	200 (0, 375)	0.004
UPDRS			
Total score	32 (23, 43)	38.5 (27, 59)	< 0.001*
UPDRS I	2 (1, 3)	2 (0, 4)	0.521
UPDRS II	9 (6, 12)	11 (7.25, 16)	< 0.001*
UPDRS III (off‐medication)	20 (13, 28)	26 (15.25, 39)	< 0.001*
Tremor score	2 (1, 4)	3 (1, 7)	< 0.001*
Rigidity score	4 (2, 6)	4 (3, 9)	< 0.001*
Bradykinesia score	9 (5, 13)	10 (5, 17.75)	0.016
PIGD score	3 (2, 4)	4 (2, 6)	< 0.001*
UPDRS IV	0 (0, 1)	0 (0, 1)	0.179
Motor subtype (%)			0.659
TD	28.13%	32.14%	
Indeterminate	8.72%	8.93%	
PIGD	63.15%	58.93%	
H&Y stage	2 (1.5, 2.5)	2.5 (1.5, 3)	< 0.001*
NMSS			
Total score	28 (14, 45)	33 (13, 58)	0.165
D1: Cardiovascular	0 (0, 1)	0 (0, 1)	0.107
D2: Sleep/fatigue	7 (2, 12)	8 (3, 16)	0.061
D3: Mood/apathy	2 (0, 8)	4 (0, 10)	0.035
D4: Perceptual problems/hallucinations	0 (0, 0)	0 (0, 0)	0.129
D5: Attention/memory	2 (0, 5)	2 (0, 6)	0.570
D6: Gastrointestinal	2 (0, 6)	2 (0, 8)	0.193
D7: Urinary function	4 (0, 8)	4 (0, 12)	0.239
D8: Sexual function	0 (0, 0)	0 (0, 0)	0.605
D9: Miscellaneous	4 (0, 8)	4 (0, 8)	0.801
MMSE	28 (26, 29)	28 (25, 29)	0.197
SCOPA‐AUT	7 (3, 11)	7 (2, 14)	0.436
HRS	24 (17, 24)	23 (16, 24)	0.403
PDSS	126 (109, 138)	124.5 (106.5, 136)	0.347
ESS	6 (2, 11.25)	8 (3, 13)	0.081
PFS	41 (32, 59)	44 (26.25, 62.75)	0.502
HAMD	4 (1, 7)	5 (2, 9)	0.061
PDQ‐39	16 (6, 31)	23 (9, 40)	0.006
Constipation (%)	26.96%	29.03%	0.672
RBD (%)	33.51%	38.95%	0.292
RLS (%)	7.92%	9.76%	0.563
LID (%)	14.06%	19.54%	0.171
FOG (%)	21.34%	30.68%	0.046

*Note:* Continuous data were displayed as median (interquartile ranges, IQR) for skewed data and categorical data were summarized by frequency (%). The significance threshold after the Bonferroni correction was *p* value < 0.0012.

Abbreviations: ESS, Epworth Sleepiness Scale; FOG, freezing of gait; H&Y stage, Hoehn and Yahr stage; HAMD, the 17‐item Hamilton Depression Rating Scale; HRS, Hyposmia Rating Scale; LEDD, levodopa equivalent daily dose; LID, levodopa‐induced dyskinesias; MMSE, Mini‐Mental State Examination; NMSS, Non‐Motor Symptoms Scale; PDQ‐39, PD Questionnaire‐39; PDSS, Parkinson's Disease Sleep Scale; PFS, Parkinson Fatigue Scale; PIGD, postural instability, and gait difficulty; RBD, rem sleep behavior disorder; RLS, restless legs syndrome; SCOPA‐AUT, Scale for Outcomes in PD for Autonomic Symptoms; TD, tremor dominant; UPDRS, Unified Parkinson's Disease Rating Scale.

* Represents a significant *p* value (*p* value < 0.0012).

Survival analysis of FOG was performed in 82 *NUS1*‐PD and 726 GU‐PD after excluding PD patients with missing FOG scales. The results of the Kaplan–Meier survival curve and the Cox proportional hazards models demonstrated no significant difference in FOG occurrence between the two groups (Table S2, Figure S2). However, a later AAO and early initiation of levodopa therapy were associated with a shorter time to FOG occurrence (HR = 1.11, 95% CI = 1.00–1.02, *p* = 0.046, and HR = 0.88, 95% CI = 0.84–0.92, *p* < 0.001, respectively).

### 3.2 Longitudinal Progression of Motor and Non‐Motor Symptoms in *NUS1* ‐PD and GU‐PD

**FIGURE 1:**
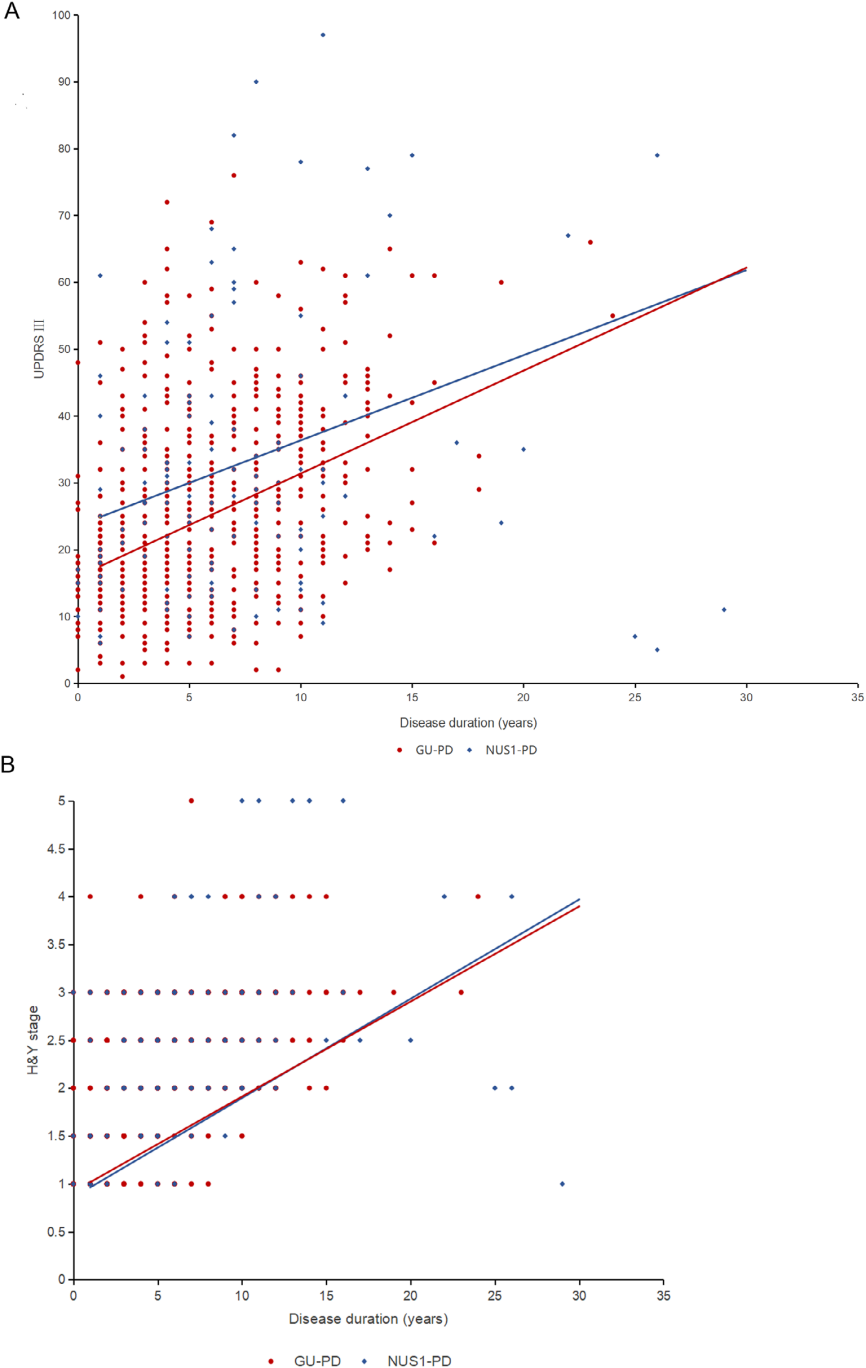
Longitudinal trajectories of UPDRS‐III (A) and H&Y stage (B) in *NUS1*‐PD (*n* = 38) and GU‐PD (*n* = 190) according to linear mixed‐effects models. H&Y, Hoehn and Yahr; UPDRS, Unified Parkinson's Disease Rating Scale.

PD patients with baseline data and at least 2 follow‐up visits were included in the longitudinal analysis, and 38 cases of *NUS1*‐PD and 190 cases of GU‐PD were incorporated to detect the relationship between *NUS1* variants and PD disease progression (Figure S1). The mean follow‐up intervals for the first and second follow‐ups were 2.50 years and 1.97 years, respectively. We found that the estimated rates of worsening in UPDRS III were 1.54 points per year in GU‐PD (SE = 0.164) and 1.27 points per year in *NUS1*‐PD (SE = 0.306) (Figure 1A). The annual deterioration rate of GU‐PD was faster than that of *NUS1*‐PD with a difference of 0.25, but the difference was not statistically significant (*p* = 0.461, Table 2). Regarding H&Y stage, *NUS1*‐PD increased by 0.104 points per year (SE = 0.016), and GU‐PD exhibited a worsening rate of 0.099 points per year (SE = 0.010) (Figure 1B). The difference between the two groups was 0.004 points, but without statistical significance (*p* = 0.814, Table 2). The results for other parts of the UPDRS scale indicated that the estimated rates of worsening were slower in *NUS1*‐PD compared to GU‐PD, except for UPDRS II, tremor score, and PIGD score, but these differences were not statistically significant (Table S3).

**TABLE 2:** Longitudinal analyses of UPDRS III and H&Y stage in two groups.

	UPDRS III (off‐medication)			H&Y stage (off‐medication)		
	*β*	95% CI	*p*	*β*	95% CI	*p*
Age at baseline (years)	0.14	−0.001, 0.28	0.052	0.02	0.01, 0.03	< 0.001*
Sex (Male = ref)	−4.06	−6.93, −1.19	0.006*	−0.12	−0.29, 0.04	0.147
Disease duration (years)	1.54	1.22, 1.86	< 0.001*	0.10	0.08, 0.12	< 0.001*
LEDD (mg/24 h)	0.001	−0.007, 0.007	0.966	0.0002	−0.0002, 0.001	0.332
Education (years)	−0.83	−1.24, −0.42	< 0.001*	−0.04	−0.06, −0.02	< 0.001*
*NUS1*‐PD	4.87	0.28, 9.46	0.038*	0.06	−0.19, 0.32	0.625
GU‐PD (ref)	—	—	—	—	—	—
Years × *NUS1*‐PD/GU‐PD	−0.25	−0.93, 0.42	0.461	0.004	−0.03, 0.04	0.814

Abbreviations: CI, confidence interval; H&Y, Hoehn and Yahr; LEDD, levodopa equivalent daily dose; ref., reference; UPDRS, Unified Parkinson's Disease Rating Scale; β, the regression coefficient.

* Represents a significant *p* value (*p* value < 0.05).

**FIGURE 2:**
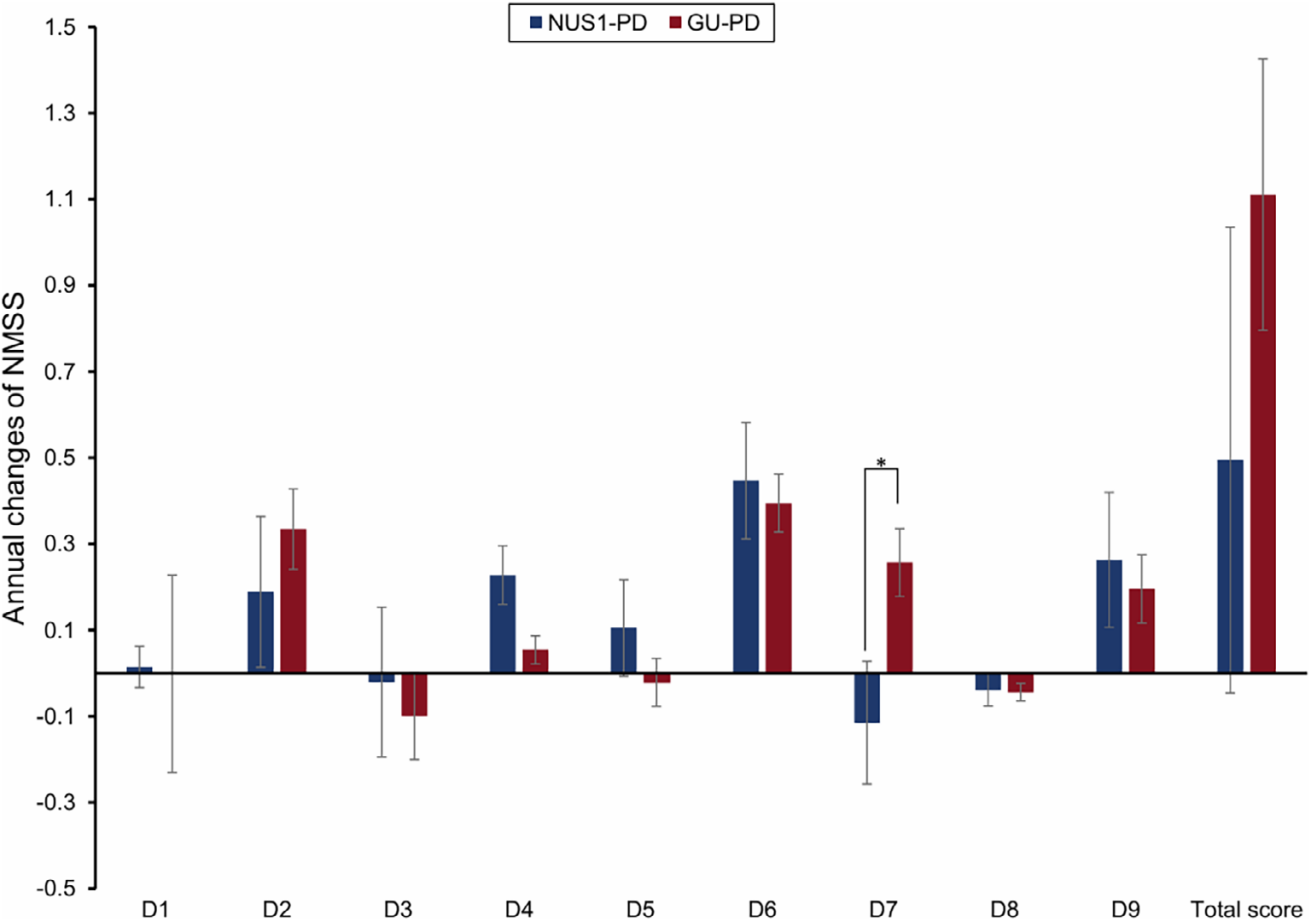
Annual changes of NMSS in two groups. The annual changes were calculated by linear mixed‐effects models, while correcting for age, gender, LEDD, UPDRS‐III, and years of education at baseline. *p < 0.05. NMSS, Non‐Motor Symptoms Scale.

The results of NMSS showed that D7 (urinary function) in GU‐PD increased with disease progression at an estimated rate of 0.26 points per year, while the score decreased at 0.12 points per year in *NUS1*‐PD. The difference between the two groups was 0.37 points and was statistically significant (*p* = 0.024, Figure 2). The disease progression rates of other components in NMSS were not statistically significant (Figure 2). Similarly, regarding other scales including MMSE, PDSS, ESS, RBDQ‐HK, HRS, PFS, SCOPA‐AUT, PDQ‐39, and HAMD, no significant differences were observed in the longitudinal analysis between *NUS1*‐PD and GU‐PD (Table S4).

### 3.3 The Correlation of Plasma NgBR Levels With PD

**FIGURE 3:**
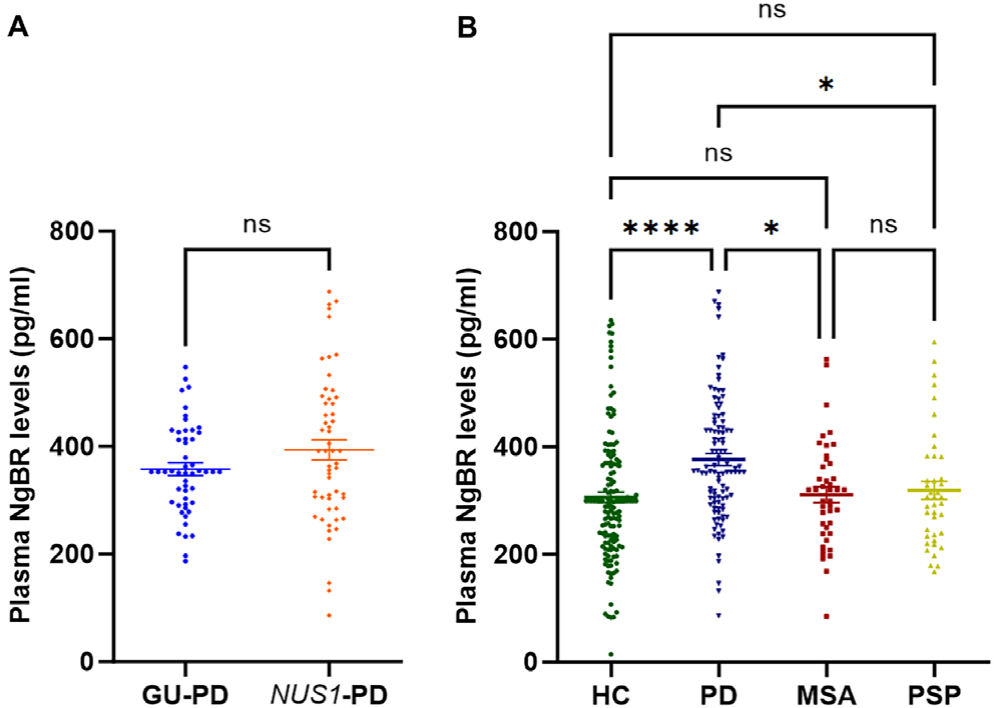
Plasma NgBR levels of participants. (A) Plasma NgBR levels of *NUS1*‐PD (*n* = 55) and GU‐PD (*n* = 51). (B) Plasma NgBR levels of HC (*n* = 147), PD (*n* = 106), MSA (*n* = 43), PSP (*n* = 41). The plasma NgBR levels were shown by scatter plots. Groups were compared with Student's t‐test and one‐way ANOVA, with the Tukey method for multiple comparison analysis test. *p < 0.05, ****p < 0.001. ns, no significance.

To investigate the association between plasma NgBR levels and PD, we included 147 cases in HC, 51 in GU‐PD, 55 in *NUS1*‐PD, 43 in MSA, and 41 in PSP for comprehensive analysis. The demographic information of all participants is displayed in Table S5. The plasma NgBR levels in *NUS1*‐PD were higher than those in GU‐PD, but they were not statistically significant (*p* = 0.104, Figure 3A). After adjusting for gender, AAO, disease duration, and plasma storage time, no significant correlation was observed between *NUS1* variant carrier status and plasma NgBR levels (*p* = 0.306, Table S6).

**FIGURE 4:**
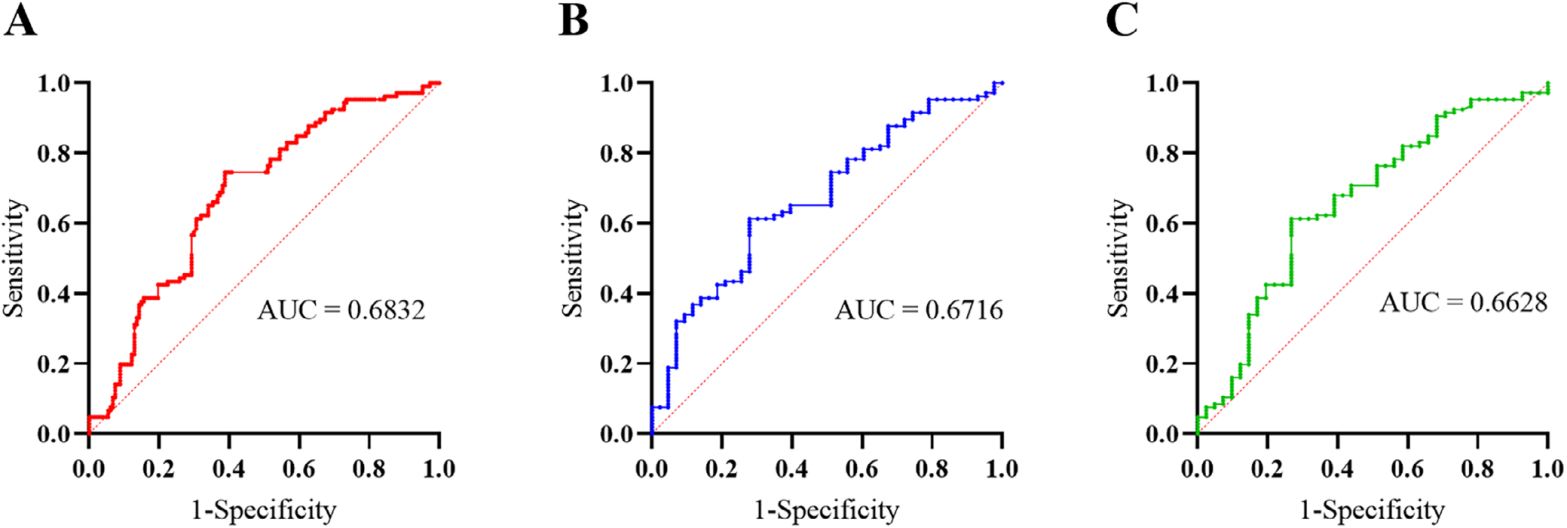
ROC curve of plasma NgBR levels in distinguishing PD from HC (A), MSA (B), and PSP (C).

In subsequent studies, we merged *NUS1*‐PD and GU‐PD into a unified PD cohort. The plasma NgBR levels of the four groups were ranked from high to low as follows: PD (376.37 ± 116.03 pg/mL), PSP (319.11 ± 107.66 pg/mL), MSA (311.17 ± 96.79 pg/mL), and HC (305.94 ± 121.74 pg/mL) (Figure 3B). There were significant differences in plasma NgBR levels among the four cohorts, which remained after adjusting for confounding factors (Table S7). The ROC curve showed that the AUC values of plasma NgBR in distinguishing PD from HC, MSA, and PSP were 0.6832, 0.6716, and 0.6628, respectively, suggesting that plasma NgBR may have the potential to assist in differentiating PD from healthy controls and Parkinson‐Plus Syndromes (Figure 4). The optimal cutoff value for differentiating PD from HC, MSA, and PSP was 341.703 pg/mL.

### 3.4 The Correlation of Plasma NgBR Levels With PD Clinical Phenotypes

We analyzed the relationship between plasma NgBR levels and clinical characteristics of PD using multivariate linear regression and logistic regression analysis methods. The results demonstrated significant correlations between plasma NgBR levels and both UPDRS total score and UPDRS III (*p* = 0.007, *p* = 0.006, respectively), with regression coefficients of 0.05 and 0.04 (Table 3). Moreover, plasma NgBR levels showed a positive correlation with the rigidity score and PIGD score of UPDRS (*p* = 0.043, *p* = 0.045, respectively). Notably, plasma NgBR levels were also associated with cognitive impairment in PD patients (*p* = 0.010), with an OR of 1.007 (Table 3).

**TABLE 3:** Correlation analysis of plasma NgBR levels and clinical characteristics of PD patients.

	*Β* (OR)	95% CI	*p*
UPDRS			
Total score	0.05	0.02, 0.09	0.007^*^
UPDRS I	−0.001	−0.004, 0.003	0.731
UPDRS II	0.010	−0.002, 0.023	0.099
UPDRS III	0.04	0.01, 0.06	0.006^*^
Tremor score	0.00	−0.01, 0.01	0.937
Rigidity score	0.007	0.00, 0.014	0.043^*^
Bradykinesia score	0.011	−0.001, 0.024	0.071
PIGD score	0.006	0.000, 0.011	0.045^*^
UPDRS IV	0.003	−0.001, 0.007	0.150
Motor subtype (%)			
TD	—	—	—
Indeterminate	1.00	0.99, 1.01	0.801
PIGD	1.00	1.00, 1.01	0.621
H&Y stage	1.00	1.00, 1.01	0.145
1.0–2.5	—	—	—
3.0–5.0	—	—	—
NMSS	−0.02	−0.07, 0.03	0.421
Total score	0.00	−0.003, 0.002	0.721
D1: Cardiovascular	0.00	−0.014, 0.013	0.952
D2: Sleep/fatigue	0.00	−0.02, 0.01	0.612
D3: Mood/apathy	−0.01	−0.01, 0.00	0.085
D4: Perceptual problems/hallucinations	0.00	−0.01, 0.01	0.919
D5: Attention/memory	−0.003	−0.012, 0.006	0.526
D6: Gastrointestinal tract	−0.01	−0.02, 0.01	0.208
D7: Urinary function	−0.004	−0.008, 0.00	0.079
D8: Sexual function	0.004	−0.006, 0.014	0.465
Constipation	0.998	0.994, 1.003	0.497
Hyposmia	1.000	0.996, 1.003	0.919
RBD	1.000	0.997, 1.004	0.990
SCOPA‐AUT	−0.001	−0.012, 0.009	0.776
PDSS	0.008	−0.026, 0.041	0.650
RLS	1.001	0.990, 1.012	0.891
EDS	1.002	0.998, 1.006	0.240
PFS	−0.007	−0.037, 0.024	0.666
PDQ‐39	0.032	−0.013, 0.077	0.159
Depression	0.999	0.995, 1.003	0.692
LID	0.999	0.994, 1.004	0.658
FOG	1.001	0.998, 1.005	0.560
CI	1.007	1.002, 1.013	0.010^*^

*Note:* Models were adjusted by sex, age at onset, disease duration and plasma storage time.

Abbreviations: CI, cognitive impairment; CI, confidence interval; FOG, freezing of gait; H&Y stage, Hoehn and Yahr stage; LID, levodopa‐induced dyskinesias; NMSS, Non‐Motor Symptoms Scale; PDQ‐39, PD Questionnaire‐39; PDSS, Parkinson's Disease Sleep Scale; PFS, Parkinson Fatigue Scale; PIGD, postural instability and gait difficulty; RBD, REM sleep behavior disorder; RLS, restless legs syndrome; excessive daytime sleepiness; SCOPA‐AUT, Scale for Outcomes in PD for Autonomic Symptoms; TD, tremor dominant; UPDRS, Unified Parkinson's Disease Rating Scale; β, the regression coefficient. ^*^Represents a significant *p* value (*p* value < 0.05).

## 4 Discussion

A growing body of research has focused on the role of the *NUS1* gene in PD. The correlation between *NUS1* and PD has been detected, and it is reported that *NUS1*‐associated PD presents unique clinical manifestations [6, 8]. However, there is currently a lack of studies on the disease progression of *NUS1*‐PD. The study prospectively observed the disease progression of *NUS1*‐PD and GU‐PD. We found that *NUS1*‐PD had an earlier AAO, a similar progression rate of motor symptoms, and a slower progression rate of urinary symptoms compared to GU‐PD. Furthermore, we also measured plasma NgBR levels in PD, PSP, MSA, and healthy controls. Our findings indicated that plasma NgBR levels were significantly higher in PD than in the other groups and were positively correlated with the severity of motor symptoms and cognitive impairment.

The median AAO of *NUS1*‐PD was 48 years, earlier than that of GU‐PD. Although the incidence of FOG in *NUS1*‐PD was higher than in GU‐PD at baseline, subsequent survival analysis failed to unveil a correlation between FOG and *NUS1* variant carrier status. Instead, we found that later AAO and earlier initiation of levodopa therapy were associated with a shorter latency to FOG appearance in PD. This aligns with prior studies suggesting that dopaminergic overstimulation and fronto‐subcortical dysfunction may contribute to FOG [27, 28, 29].

The study utilized linear mixed‐effects models to adjust for confounding factors and analyzed the impact of *NUS1* variants on PD symptom progression. The annual worsening rate of UPDRS III (off‐medication) was 1.27 in *NUS1*‐PD and 1.54 in GU‐PD, respectively. The progression rate of *NUS1*‐PD was slightly slower than that of GU‐PD, but the difference did not achieve statistical significance. Previous studies have indicated that the progression rates of UPDRS III in PD range from approximately 0.6 to 1.5 points per year, similar to this study [30, 31, 32]. Additionally, there was no significant difference in the longitudinal changes of H&Y stage between *NUS1*‐PD and GU‐PD, with an annual change of approximately 0.1 points, consistent with previous studies [33].

Although longitudinal progression rates were comparable, baseline UPDRS III and H&Y stage were significantly higher in *NUS1*‐PD, even after adjusting for disease duration. Linear mixed‐effects models calculate the average annual change over the entire follow‐up period for PD patients, but the disease progression of PD is known to be non‐linear. Progression rates of PD vary across different disease stages, with faster changes in later stages (up to 3.96 points per year) and slower in early stages (0.67 points per year) [34]. Therefore, we speculate that *NUS1*‐PD may progress more rapidly in the early disease stage compared to GU‐PD. As PD advances, the progression rate of *NUS1*‐PD may gradually become similar to that of GU‐PD.

Regarding non‐motor symptoms, we observed that urinary function declined more slowly in *NUS1*‐PD than in GU‐PD. No significant group differences were found in the progression of other non‐motor symptoms. Prior investigations have indicated that urinary function in PD deteriorates with disease progression, a trend that is consistent with the GU‐PD in our study. Conversely, in the *NUS1*‐PD, urinary function gradually ameliorates as the disease progresses. The urinary function of PD patients is associated with central nervous system involvement (e.g., basal ganglia, frontal cortex, insula) and α‐synuclein aggregation in peripheral autonomic neurons [35, 36]. Our findings suggest that urinary function impairment in *NUS1*‐PD is less severe, but the specific pathological mechanisms require further investigation.

We measured plasma NgBR levels and elucidated no significant differences in plasma NgBR levels between *NUS1*‐PD and GU‐PD. *NUS1* variants may not directly influence NgBR expression in plasma. Potential explanations for these results are as follows: The study included PD patients carrying nonsynonymous variants in the coding region of the *NUS1* gene. Most of these variants were rare (MAF < 0.01), and all variant carriers were pooled together for joint analysis in *NUS1*‐PD. Different variants in the *NUS1* gene may exert opposite effects on *NUS1* expression, which could lead to false‐negative outcomes.

Nonetheless, we found that plasma NgBR levels were significantly higher in PD compared to healthy controls, MSA, and PSP. Previous research indicates that NgBR plays an important role in lysosomal cholesterol transport by interacting with NPC2, a cholesterol‐binding protein [13, 37]. NgBR deficiency can impair lysosomal function, promote α‐synuclein toxicity, and reduce dopaminergic neurons [12]. So, NgBR dysfunction may contribute to PD by modulating cholesterol efflux in the lysosome and α‐syn neurotoxicity. However, NgBR regulating lysosomal cholesterol efflux is independent of Nogo‐B (the interaction protein of NgBR), and the increase of NgBR has little effect on Nogo‐B expression [37, 38]. These mechanisms suggest a protective role for NgBR in PD. However, paradoxically, plasma NgBR levels were increased in PD. The elevated level of plasma NgBR in PD may be attributed to the inhibition of intracellular protein interactions leading to PD, which in turn increases plasma NgBR in a feedback manner. This upregulation may serve to modulate NPC2 activity, reduce cholesterol synthesis, and relieve the pathological changes of PD [39]. Similar to α‐syn, NgBR may have peripheral sources contributing to its plasma concentration. Given the blood–brain barrier, plasma levels may not fully reflect changes in the central nervous system. Measuring NgBR in CSF will be critical to clarify its relevance in PD.

The diagnosis of PD is challenging due to similar clinical symptoms to Parkinson‐plus syndromes. Therefore, it is crucial to explore biomarkers for the diagnosis of PD. ROC curve analysis revealed that plasma NgBR levels may assist in distinguishing PD from healthy controls, MSA, and PSP, with the AUC values range of 0.6–0.7. Further research could combine NgBR with other biomarkers to construct diagnostic models to improve diagnostic accuracy. Besides, plasma NgBR levels were positively correlated with UPDRS total and III scores, as well as cognitive impairment. However, the causal relationship and underlying mechanisms between plasma NgBR levels and clinical features remain unclear and require further studies for elucidation.

Our study has several limitations that need to be addressed in future research. First, the longitudinal study of disease progression included only 38 *NUS1*‐PD patients. The statistical power for linear mixed‐effects models was calculated using GLIMMPSE (version 3.1.3, https://glimmpse.samplesizeshop.org/), yielding results exceeding 0.8. However, the relatively small sample size (*n* = 38) may reduce the power of detecting small‐to‐moderate effects, especially in subgroup comparisons. Further research should expand the sample size, shorten the follow‐up intervals, and extend the follow‐up duration to reduce bias. Second, we utilized linear mixed‐effects models to obtain the annual change of scales, but PD progression is not linear. Finally, although we detected that plasma NgBR levels could potentially assist in PD diagnosis, we must be cautious of the potential bias introduced by the small sample size and single‐center study. Plasma NgBR levels were unable to represent the changes in NgBR of the central nervous system. To further explore the pathogenic mechanism of NgBR and improve the diagnostic efficacy, multi‐center and large‐sample studies in detecting plasma and CSF NgBR are required.

## 5 Conclusion

In summary, longitudinal analyses of clinical symptoms in the Chinese population show similar motor progression in *NUS1*‐PD compared with GU‐PD. Moreover, our results suggest that plasma NgBR levels were elevated in PD and correlated with motor and cognitive impairments, suggesting potential as a biomarker. Our findings support the involvement of *NUS1* and NgBR in PD pathogenesis. Nevertheless, more extensive and longer longitudinal studies and pathogenesis mechanism research are warranted to verify our results.

## Author Contributions

**Lizhi Li:** writing – original draft; conceptualization; data curation; investigation; methodology; formal analysis. **Juanjuan Huang:** writing – original draft; investigation. **Yaqin Xiang, Xuxiang Zhang, Qian Xu, Qiying Sun, Zhenhua Liu, and Xinxiang Yan:** investigation. **Jinchen Li:** funding acquisition. **Beisha Tang:** conceptualization; supervision; methodology. **Jifeng Guo:** conceptualization; methodology; investigation; supervision; writing – original draft; funding acquisition. All authors read and approve the final manuscript.

## Ethics Statement

All participants recruited in the study have written informed consent, and the study was approved by Xiangya Hospital of Central South University. The study was performed in accordance with the ethical standards laid down in the 1964 Declaration of Helsinki and its later amendments.

## Conflicts of Interest

The authors declare no conflicts of interest.

## Supporting information


**Table S1:** Correlation analysis of *NUS1* variants with baseline UPDRS scores and H&Y stage.
**Table S2:**. Cox proportional hazards models for freezing of gait.
**Table S3:** Longitudinal analyses of other UPDRS scales in *NUS1*‐PD and GU‐PD.
**Table S4:** Longitudinal analyses of other non‐motor scales between *NUS1*‐PD and GU‐PD.
**Table S5:** Demographic and clinical characteristics of participants in plasma NgBR study.
**Table S6:** Correlation analysis of *NUS1* variants and plasma NgBR levels in PD patients.
**Table S7:** Correlation analysis of plasma NgBR levels with PD, HC, and Parkinson‐Plus Syndromes.
**Table S8:** Demographic and clinical characteristics of PD patients without other PD known pathogenic genes at baseline.
**Table S9:** The specific information of *NUS1* variants in the study.
**Table S10:** The results of power calculated by GLIMMPSE.
**Figure S1:** Flow diagram of study participation. Abbreviations: PD‐MDCNC, Parkinson’s Disease & Movement Disorders Multicenter Database and Collaborative Network in China.
**Figure S2:** Kaplan–Meier curves for time to occurrence of FOG. Log‐rank tests were performed to compare the survival curves between GU‐PD and *NUS1*‐PD. Abbreviations: FOG, freezing of gait.
**Figure S3:** The plasma NgBR levels in PD patients with and without PD medication. The plasma NgBR levels of “on‐medication” and “off‐medicaiton” were compared by Student’s t‐test in all PD patients (on‐medication, *n* = 32; off‐medication, *n* = 74) (A), *NUS1*‐PD (on‐medication, *n* = 14; off‐medication, *n* = 41) (B), and GU‐PD (on‐medication, *n* = 18; off‐medication, *n* = 33) (C). Significant threshold: *p* < 0.05. ns, no significance.

## Data Availability

The data supporting this study's findings are available from the corresponding author upon reasonable request.
