# Supplementary material for: The Progression of NUS1 ‐Associated Parkinson's Disease and the Diagnostic Potential of Plasma NgBR
Source: CNS Neurosci Ther. 2025 Jul 28;31(7):e70549. doi: 10.1111/cns.70549 (PMC12304428; doi:10.1111/cns.70549)

**Supplementary Table 1.** Correlation analysis of *NUS1* variants with baseline UPDRS scores and H&Y stage

|  | β | 95%CI | *p* |
| --- | --- | --- | --- |
| UPDRS total score | 7.30 | 3.85, 10.75 | **<0.001** |
| UPDRS I | 0.18 | -0.22, 0.58 | 0.374 |
| UPDRS II | 1.75 | 0.74, 2.76 | **<0.001** |
| UPDRS III (off-medication) | 5.13 | 2.68, 7.58 | **<0.001** |
| Tremor score | 1.41 | 0.80, 2.02 | **<0.001** |
| Rigidity score | 1.08 | 0.33, 1.83 | **0.005** |
| Bradykinesia score | 1.37 | 0.18, 2.55 | **0.024** |
| PIGD score | 1.06 | 0.61, 1.50 | **<0.001** |
| UPDRS IV | 0.24 | -0.14, 0.61 | 0.220 |
| H&Y stage | 0.25 | 0.12, 0.39 | **<0.001** |

Abbreviations: β, the regression coefficient; CI, confidence interval; UPDRS, Unified Parkinson's Disease Rating Scale; PIGD, postural instability and gait difficulty; H&Y stage, Hoehn and Yahr stage.

The statistically significant *p* was presented in bold text.

**Supplementary Table 2.** Cox proportional hazards models for freezing of gait

|  | Univariable | | Multivariable | |
| --- | --- | --- | --- | --- |
|  | HR (95%CI) | *p* | HR (95%CI) | *p* |
| *NUS1*-PD | 0.94 (0.69, 1.27) | 0.670 | 1.11 (0.82, 1.50) | 0.509 |
| GU-PD (ref) | - | - | - | - |
| Age at onset | - | - | 1.01 (1.00, 1.02) | **0.046** |
| Sex (male=ref) | - | - | 0.84 (0.68, 1.02) | 0.835 |
| Disease duration at levodopa therapy initiation | - | - | 0.88 (0.84, 0.92) | **<0.001** |

Abbreviations: HR, Hazard ratio; CI, confidence interval; ref, reference.

The statistically significant *p* was presented in bold text.

**Supplementary Table 3.** Longitudinal analyses of other UPDRS scales in *NUS1*-PD and GU-PD

|  | β | 95%CI | *p* |
| --- | --- | --- | --- |
| UPDRS total score | -0.21 | -1.16, 0.75 | 0.669 |
| UPDRS I | -0.02 | -0.10, 0.06 | 0.616 |
| UPDRS II | 0.04 | -0.24, 0.33 | 0.759 |
| Tremor score | 0.14 | -0.04, 0.32 | 0.133 |
| Rigidity score | -0.10 | -0.31, 0.11 | 0.361 |
| Bradykinesia score | -0.25 | -0.57, 0.07 | 0.124 |
| PIGD score | 0.02 | -0.14, 0.19 | 0.749 |
| UPDRS IV | -0.01 | -0.42, 0.41 | 0.926 |

Abbreviations: β, the regression coefficient; CI, confidence interval; UPDRS, Unified Parkinson's Disease Rating Scale; PIGD, postural instability and gait difficulty.

The statistically significant *p* was presented in bold text.

**Supplementary Table 4.** Longitudinal analyses of other non-motor scales between *NUS1*-PD and GU-PD

|  | GU-PD | | *NUS1*-PD | | Years ×  *NUS1*-PD/GU-PD | 95%CI | *p* |
| --- | --- | --- | --- | --- | --- | --- | --- |
|  | Annual changes | SE | Annual changes | SE |  |  |  |
| MMSE | -0.07 | 0.040 | -0.09 | 0.075 | -0.02 | -0.19, 0.15 | 0.795 |
| PDSS | -0.16 | 0.298 | 0.48 | 0.460 | 0.63 | -0.44, 1.71 | 0.246 |
| ESS | 0.25 | 0.079 | 0.02 | 0.152 | -0.23 | -0.57, 0.12 | 0.192 |
| RBDQ-HK | 0.97 | 0.219 | 0.70 | 0.412 | -0.27 | -1.19, 0.65 | 0.563 |
| HRS | -0.35 | 0.094 | -0.46 | 0.180 | -0.10 | -0.50, 0.30 | 0.611 |
| PFS | -0.03 | 0.256 | -0.43 | 0.349 | -0.39 | -1.24, 0.45 | 0.361 |
| SCOPA-AUT | 0.42 | 0.094 | 0.15 | 0.168 | -0.27 | -0.65, 0.11 | 0.161 |
| PDQ39 | 1.49 | 0.276 | 1.54 | 0.484 | 0.05 | -1.03, 1.14 | 0.921 |
| HAMD | 1.65 | 1.310 | -0.07 | 1.854 | -1.72 | -6.17, 2.73 | 0.448 |

Abbreviations: SE, standard error; CI, confidence interval; MMSE, Mini-Mental State Examination; PDSS, Parkinson’s Disease Sleep Scale; ESS, Epworth Sleepiness Scale; RBDQ-HK, Rapid Eye Movement Sleep Behavior Disorder Questionnaire-Hong Kong; HRS, Hyposmia Rating Scale; PFS, Parkinson Fatigue Scale; SCOPA-AUT, Scale for Outcomes in PD for Autonomic Symptoms; PDQ39, Parkinson's Disease Questionnaire-39; HAMD, 17-item Hamilton Depression Rating Scale.

The statistically significant *p* was presented in bold text.

**Supplementary Table 5.** Demographic and clinical characteristics of participants in plasma NgBR study

|  | HC  (n=147) | GU-PD  (n=51) | *NUS1*-PD  (n=55) | MSA  (n=43) | PSP  (n=41) | *p* |
| --- | --- | --- | --- | --- | --- | --- |
| Age, y | 53.57±14.44 | 59.35±10.05 | 58.96±11.61 | 58.19±8.07 | 64.83±6.84 | **<0.001** |
| Male (%) | 50.34% | 47.06% | 60.00% | 60.46% | 53.66% | 0.527 |
| Age at onset, y | - | 51.57±10.12 | 49.84±11.25 | 55.60±8.00 | 62.32±6.79 | **<0.001** |
| Disease duration, y | - | 8.60±5.61 | 9.72±5.57 | 2.99±2.20 | 3.21±1.62 | **<0.001** |
| Plasma storage time, y | 1.80±0.34 | 1.62±0.52 | 0.97±0.58 | 1.28±0.63 | 1.02±0.71 | **<0.001** |

Continuous data were displayed as mean ± SD and categorical data were summarized by frequency (%). The statistically significant *p* was presented in bold text.

**Supplementary Table 6.** Correlation analysis of *NUS1* variants and plasma NgBR levels in PD patients.

|  | Plasma NgBR levels | | | |
| --- | --- | --- | --- | --- |
|  | β | 95%CI | Beta | *p* |
| *NUS1*-PD | 26.57 | -24.72, 77.86 | 0.12 | 0.306 |
| GU-PD (ref) | - | - | - | - |
| Sex (male=ref) | 25.37 | -19.72, 70.46 | 0.11 | 0.267 |
| Age at onset, y | -2.37 | -4.50, -0.24 | -0.22 | **0.030** |
| Disease duration, y | -0.00 | -4.07, 4.07 | 0.00 | 1.000 |
| Plasma storage time, y | -13.37 | -54.77, 28.03 | -0.07 | 0.523 |

Abbreviations: β, the regression coefficient; CI, confidence interval; Beta, the standardized regression coefficients.

The statistically significant *p* was presented in bold text.

**Supplementary Table 7.** Correlation analysis of plasma NgBR levels with PD, HC, and Parkinson-Plus Syndromes

|  | Plasma NgBR levels | | | |
| --- | --- | --- | --- | --- |
|  | β | 95%CI | Beta | *p* |
| PSP | -56.68 | -97.94, -15.41 | -0.16 | **0.007** |
| MSA | -65.28 | -105.11, -25.35 | -0.18 | **0.001** |
| HC | -63.93 | -94.51, -33.35 | -0.27 | **<0.001** |
| PD（ref） | - | - | - | - |
| Sex (male=ref) | 24.09 | -0.12, 48.29 | 0.10 | 0.051 |
| Age, y | -1.74 | -2.75, -0.72 | -0.18 | **<0.001** |
| Plasma storage time, y | -35.63 | -58.24, -13.02 | -0.18 | **0.002** |

Abbreviations: β, the regression coefficient; CI, confidence interval; Beta, the standardized regression coefficients.

The statistically significant *p* was presented in bold text.

**Supplementary Table 8.** Demographic and clinical characteristics of PD patients without other PD known pathogenic genes at baseline.

|  | GU-PD  (n=768) | *NUS1*-PD^#^  (n=85) | *p* |
| --- | --- | --- | --- |
| Demographics |  |  |  |
| Male (%) | 52.08% | 58.82% | 0.238 |
| Year of education, y | 10 (6, 12) | 9 (6, 12) | 0.348 |
| Age at onset, y | 54 (47, 61) | 49 (43, 57.5) | **0.001**** |
| Age at baseline, y | 58 (51, 65) | 55 (47, 64.5) | 0.054 |
| Disease duration, y | 2 (1, 5) | 3 (1, 7) | **0.006*** |
| Disease duration at levodopa therapy initiation, y | 1 (0, 2) | 1 (0, 3) | **0.001**** |
| LEDD, mg/24h | 300 (150, 450) | 200 (0, 375) | **0.011*** |
| UPDRS |  |  |  |
| Total score | 32 (23, 43) | 37 (27, 58.5) | **0.001**** |
| UPDRS I | 2 (1, 3) | 2 (0, 4) | 0.663 |
| UPDRS II | 9 (6, 12, | 11 (7.5, 16) | **0.002*** |
| UPDRS III (off-medication) | 20 (13, 28) | 26 (15.5, 37.5) | **0.002*** |
| Tremor score | 2 (1, 4) | 3 (1.5, 7.5) | **<0.001**** |
| Rigidity score | 4 (2, 6) | 4 (3, 8) | **0.010*** |
| Bradykinesia score | 9 (5, 13) | 10 (5, 17.5) | **0.027*** |
| PIGD score | 3 (2, 4) | 4 (2, 6) | **<0.001**** |
| UPDRS IV | 0 (0, 1) | 0 (0, 1.5) | 0.270 |
| Motor subtype (%) |  |  | 0.366 |
| TD | 28.13% | 34.12% |  |
| Indeterminate | 8.72% | 10.59% |  |
| PIGD | 63.15% | 55.29% |  |
| H&Y stage | 2 (1.5, 2.5) | 2.5 (1.5, 3) | **0.002*** |
| NMSS |  |  |  |
| Total score | 28 (14, 45) | 30 (12, 60.25) | 0.352 |
| D1: Cardiovascular | 0 (0, 1) | 0 (0, 1) | 0.211 |
| D2: Sleep/fatigue | 7 (2, 12) | 8 (3, 17) | 0.079 |
| D3: Mood/apathy | 2 (0, 8) | 4 (0, 11.25) | 0.077 |
| D4: Perceptual problems/hallucinations | 0 (0, 0) | 0 (0, 0) | 0.282 |
| D5: Attention/memory | 2 (0, 5) | 2 (0, 6) | 0.505 |
| D6: Gastrointestinal | 2 (0, 6) | 2 (0, 8) | 0.396 |
| D7: Urinary function | 4 (0, 8) | 3.5 (0, 12) | 0.414 |
| D8: Sexual function | 0 (0, 0) | 0 (0, 0) | 0.365 |
| D9: Miscellaneous | 4 (0, 8) | 4 (0, 8) | 0.886 |
| MMSE | 28 (26, 29) | 28 (25, 29) | 0.152 |
| SCOPA-AUT | 7 (3, 11) | 4 (2, 14) | 0.987 |
| HRS | 24 (17, 24) | 24 (13, 24) | 0.716 |
| PDSS | 126 (109, 138) | 122.5 (104.5, 136.25) | 0.335 |
| ESS | 6 (2, 11.25) | 8 (3, 13) | **0.043*** |
| PFS | 41 (32, 59) | 44 (22.75, 63.75) | 0.604 |
| HAMD | 4 (1, 7) | 5.5 (2, 9) | 0.058 |
| PDQ-39 | 16 (6, 31) | 25 (10, 41.5) | **0.003*** |
| Constipation (%) | 26.96% | 23.94% | 0.582 |
| RBD (%) | 33.51% | 39.73% | 0.284 |
| RLS (%) | 7.92% | 12.70% | 0.187 |
| LID (%) | 14.06% | 20.00% | 0.178 |
| FOG (%) | 21.34% | 32.35% | **0.037*** |

Abbreviations: *NUS1*-PD^#^, the cohort *NUS1*-PD excluding PD patients with other known PD pathogenic genes, LEDD, levodopa equivalent daily dose; UPDRS, Unified Parkinson's Disease Rating Scale; PIGD, postural instability and gait difficulty; TD, tremor dominant; H&Y stage, Hoehn and Yahr stage; NMSS, Non-Motor Symptoms Scale; MMSE, Mini-Mental State Examination; SCOPA-AUT, Scale for Outcomes in PD for Autonomic Symptoms; HRS, Hyposmia Rating Scale; PDSS, Parkinson’s Disease Sleep Scale; ESS, Epworth Sleepiness Scale; PFS, Parkinson Fatigue Scale; HAMD, the 17-item Hamilton Depression Rating Scale; PDQ-39, PD Questionnaire-39; RBD, rem sleep behavior disorder; RLS, restless legs syndrome; LID, levodopa-induced dyskinesias; FOG, freezing of gait.

Continuous data were displayed as median (interquartile ranges, IQR) for skewed data and categorical data were summarized by frequency (%). The significance threshold after the Bonferroni correction was *p* value < 0.0012. * represents a suggestively significant *p* value (*p* value < 0.05) and ** represents a significant *p* value (*p* value < 0.0012).

**Supplementary Table 9.** The specific information of *NUS1* variants in the study.

| Nucleotide change | AA change | Number of carriers | Function | SIFT | Mutation Taster | CADD | PolyPhen-2 |
| --- | --- | --- | --- | --- | --- | --- | --- |
| c.64C>T | p.L22F | 1 | nonsynonymous | T | D | D(24.6) | D(0.997) |
| c.107T>C | p.I36T | 1 | nonsynonymous | D | D | D(20.4) | B(0.010) |
| c.108C>G | p.I36M | 1 | nonsynonymous | D | D | D(23.0) | D(0.875) |
| c.174G>T | p.K58N | 1 | nonsynonymous | D | B | D(21.4) | B(0.244) |
| c.197G>C | p.R66P | 31 | nonsynonymous | T | B | D(20.4) | B(0.001) |
| c.199C>G | p.R67G | 1 | nonsynonymous | D | D | D(20.6) | B(0.057) |
| c.230G>A | p.C77Y | 1 | nonsynonymous | D | B | T(9.435) | B(0.001) |
| c.257G>T | p.R86L | 3 | nonsynonymous | D | B | D(24.7) | D(1.000) |
| c.265G>T | p.A89S | 1 | nonsynonymous | T | B | T(18.89) | B(0.001) |
| c.271G>T | p.G91C | 1 | nonsynonymous | D | B | D(22.4) | B(0.027) |
| c.392A>G | p.Y131C | 1 | nonsynonymous | T | D | D(25.2) | D(0.998) |
| c.432T>G | p.N144K | 2 | nonsynonymous | D | D | D(22.6) | D(0.777) |
| c.475C>A | p.L159I | 1 | nonsynonymous | T | D | D(26.1) | D(0.923) |
| c.487G>C | p.D163H | 7 | nonsynonymous | D | B | D(28.4) | D(1.000) |
| c.514G>A | p.A172T | 1 | nonsynonymous | T | B | T(17.64) | B(0.257) |
| c.537T>A | p.D179E | 51 | nonsynonymous | T | B | T(16.21) | B(0.001) |
| c.627G>C | p.Q209H | 2 | nonsynonymous | T | D | T(15.02) | B(0.069) |
| c.697A>G | p.N233D | 1 | nonsynonymous | T | B | T(15.47) | B(0.001) |
| c.718T>A | p.L240I | 1 | nonsynonymous | D | B | D(23.1) | D(0.649) |
| c.790G>A | p.V264I | 1 | nonsynonymous | T | D | T(2.985) | B(0.001) |
| c.882+15A>T | - | 2 | nonsynonymous | - | - | - | - |

Abbreviations: B, Benign; D, Deleterious; T, Tolerable. The variant with PHRED score≥20 was considered deleterious in CADD.

**Supplementary Table 10.** The results of power calculated by GLIMMPSE

|  | test | power | number of repeated measures | lower bound | upper bound | means scale factor | variability scale factor |
| --- | --- | --- | --- | --- | --- | --- | --- |
| UPDRS Ⅲ | Hotelling Lawley Trace | 0.882 | 3 | null | null | 2 | 1 |
| H&Y stage | Hotelling Lawley Trace | 0.881 | 3 | null | null | 2 | 1 |

**Supplementary Fig 1.** Flow diagram of study participation. Abbreviations: PD-MDCNC, Parkinson’s Disease & Movement Disorders Multicenter Database and Collaborative Network in China.

**
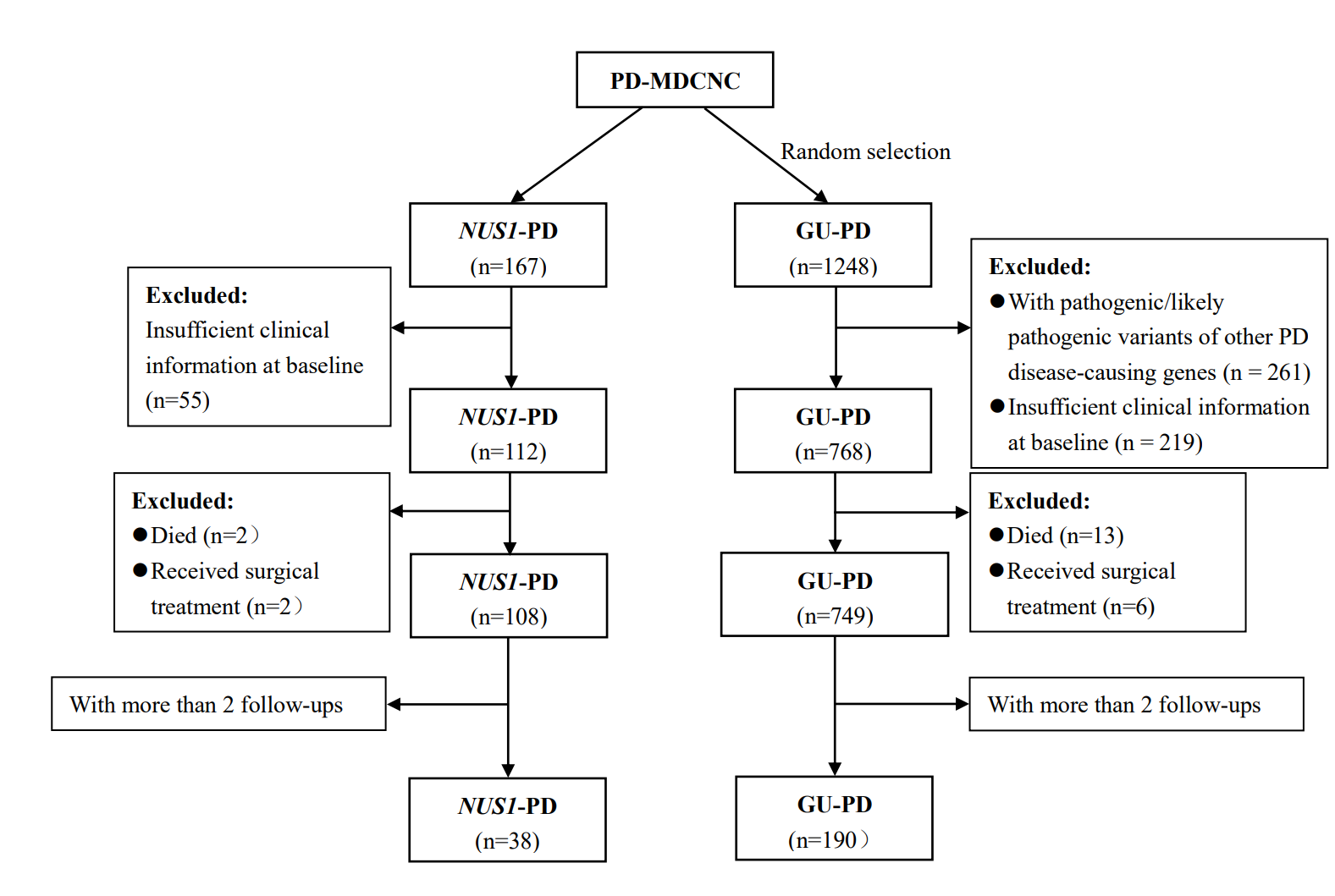
**

**Supplementary Fig 2.** Kaplan-Meier curves for time to occurrence of FOG. Log-rank tests were performed to compare the survival curves between GU-PD and *NUS1*-PD. Abbreviations: FOG, freezing of gait.

**Supplementary Fig 3.** The plasma NgBR levels in PD patients with and without PD medication. The plasma NgBR levels of “on-medication” and “off-medicaiton” were compared by Student's t-test in all PD patients (on-medication, n = 32; off-medication, n = 74) (A) , *NUS1*-PD (on-medication, n = 14; off-medication, n = 41) (B), and GU-PD (on-medication, n = 18; off-medication, n = 33) (C). Significant threshold: p<0.05. Abbreviations: ns, no significance.


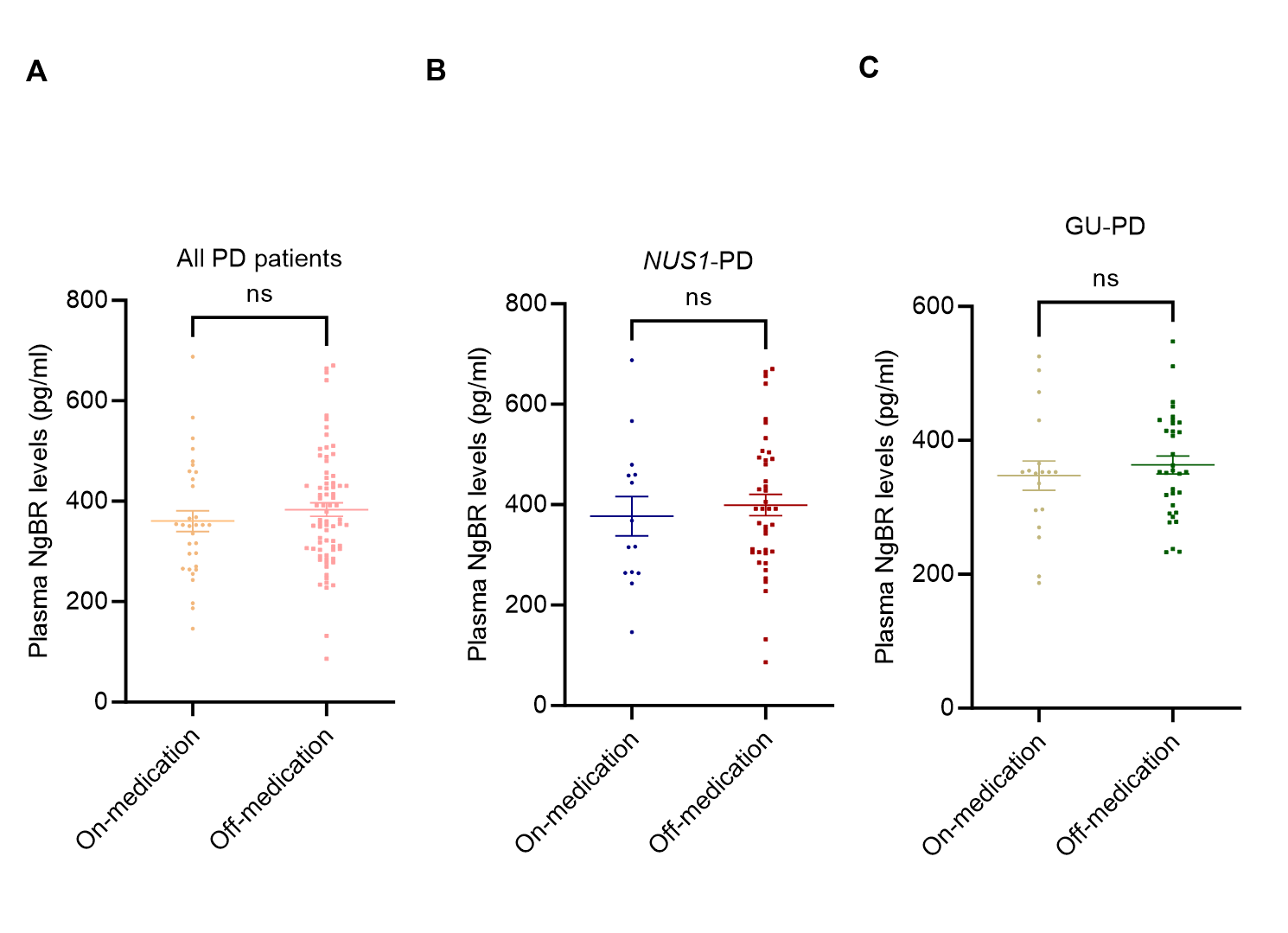

Supplement: Supplementary file 1 — Table S1: Correlation analysis of NUS1 variants with baseline UPDRS scores and H&Y stage. Table S2:. Cox proportional hazards models for freezing of gait. Table S3: Longitudinal analyses of other UPDRS scales in NUS1‐PD and GU‐PD. Table S4: Longitudinal analyses of other non‐motor scales between NUS1‐PD and GU‐PD. Table S5: Demographic and clinical characteristics of participants in plasma NgBR study. Table S6: Correlation analysis of NUS1 variants and plasma NgBR levels in PD patients. Table S7: Correlation analysis of plasma NgBR levels with PD, HC, and Parkinson‐Plus Syndromes. Table S8: Demographic and clinical characteristics of PD patients without other PD known pathogenic genes at baseline. Table S9: The specific information of NUS1 variants in the study. Table S10: The results of power calculated by GLIMMPSE. Figure S1: Flow diagram of study participation. Abbreviations: PD‐MDCNC, Parkinson’s Disease & Movement Disorders Multicenter Database and Collaborative Network in China. Figure S2: Kaplan–Meier curves for time to occurrence of FOG. Log‐rank tests were performed to compare the survival curves between GU‐PD and NUS1‐PD. Abbreviations: FOG, freezing of gait. Figure S3: The plasma NgBR levels in PD patients with and without PD medication. The plasma NgBR levels of “on‐medication” and “off‐medicaiton” were compared by Student’s t‐test in all PD patients (on‐medication, n = 32; off‐medication, n = 74) (A), NUS1‐PD (on‐medication, n = 14; off‐medication, n = 41) (B), and GU‐PD (on‐medication, n = 18; off‐medication, n = 33) (C). Significant threshold: p < 0.05. ns, no significance. [file CNS-31-e70549-s001.docx]
